# Recent Insights Into SREBP as a Direct Mediator of Kidney Fibrosis via Lipid-Independent Pathways

**DOI:** 10.3389/fphar.2020.00265

**Published:** 2020-03-17

**Authors:** Debra Dorotea, Daisuke Koya, Hunjoo Ha

**Affiliations:** ^1^Graduate School of Pharmaceutical Sciences, College of Pharmacy, Ewha Womans University, Seoul, South Korea; ^2^Department of Internal Medicine, Kanazawa Medical University, Ishikawa, Japan

**Keywords:** SREBP, TGFβ, lipotoxicity, renal lipid, kidney fibrosis

## Abstract

Sterol regulatory-element binding proteins (SREBPs) are classical regulators of cellular lipid metabolism in the kidney and other tissues. SREBPs are currently recognized as versatile transcription factors involved in a myriad of cellular processes. Meanwhile, SREBPs have been recognized to mediate lipotoxicity, contributing to the progression of kidney diseases. SREBP1 has been shown to bind to the promoter region of TGFβ, a major pro-fibrotic signaling mechanism in the kidney. Conversely, TGFβ activates SREBP1 transcriptional activity suggesting a positive feedback loop of SREBP1 in TGFβ signaling. Public ChIP-seq data revealed numerous non-lipid transcriptional targets of SREBPs that plausibly play roles in progressive kidney disease and fibrosis. This review provides new insights into SREBP as a mediator of kidney fibrosis via lipid-independent pathways.

## Introduction

Numerous studies have revealed the molecular mechanisms of sterol regulatory-element binding proteins (SREBPs) as transcription factors that critically regulate lipid homeostasis (Brown and Goldstein, 1997). The extended role of SREBPs within the last three decades has been attributed to its versatility in integrating multiple cellular signals to control, not only lipogenesis but also unexpected pathways that are important for diverse biological processes, such as endoplasmic reticulum (ER) stress, inflammation, autophagy, and apoptosis. Accordingly, SREBPs contribute to the pathogenesis of various diseases, such as diabetes mellitus, fatty liver disease, chronic kidney disease (CKD), neurodegenerative diseases, and cancers (Shimano and Sato, 2017).

CKD, a major public health problem in many countries (Webster et al., 2017), is defined by persistent urine abnormalities, structural abnormalities or impaired excretory renal function, which suggests a loss of functional nephrons (Romagnani et al., 2017). To date, renin-angiotensin system inhibitors are the mainstay of therapeutic options available to reduce albuminuria and slow the progression of CKD. However, these drugs show limitations in delaying the onset of kidney fibrosis, a common feature of end-stage kidney disease (Breyer and Susztak, 2016). Therefore, it remains interesting to explore the important target that mediates the disease progression, which can be further exploited to develop novel disease-modifying therapies.

Various factors, such as infiltrating immune cells, albuminuria, and glucosuria in diabetes, have been well recognized to activate proximal tubular epithelial cells, resulting in the secretion of pro-inflammatory and pro-fibrotic mediators that further promote interstitial inflammation and fibrosis development (Romagnani et al., 2017). Meanwhile, lipid accumulation in tubular cells has been associated with an increase in reactive oxygen species production, the loss of ATP production, apoptosis, and elevated inflammatory cytokines, which contribute to the development of tissue fibrosis (Simon and Hertig, 2015). In earlier studies, SREBP1 activation was shown to induce lipotoxicity that consequently extended SREBP-related pathology to include inflammation and fibrosis in the kidney (Sun et al., 2002). SREBP1 was further revealed to bind to the promoter region of transforming growth factor (TGF)β, a major pro-fibrotic signaling mechanism in the kidney (Uttarwar et al., 2012). Thus, SREBPs appear to mediate the progression of CKD via both lipid-dependent and -independent pathways. This review summarized the recent findings involving the TGFβ signaling pathway as a regulatory target of SREBP and predicts other putative target genes of SREBP in mediating kidney fibrosis.

## SREBPs as Classic Mediators of Lipotoxicity

### The SREBP Family

SREBPs are a family of membrane-bound transcription factors involved in lipid homeostasis. Three isoforms of SREBPs, SREBP1a, -1c, and -2, are encoded by two different genes. SREBP1a and -1c originate from different promotors of sterol regulatory element-binding transcription factor (SREBF)1 genes, whereas SREBP2 is derived from the SREBF2 gene (Goldstein et al., 2006). SREBP1a stimulates global lipid synthesis in proliferating cells, and SREBP1c plays a major role in the nutritional regulation of fatty acid and triglyceride (TG) synthesis in lipogenic organs, such as the liver. In contrast, SREBP2 ubiquitously regulates sterol synthesis in tissues (Shimano et al., 1997; Horton et al., 2002).

**FIGURE 1:**
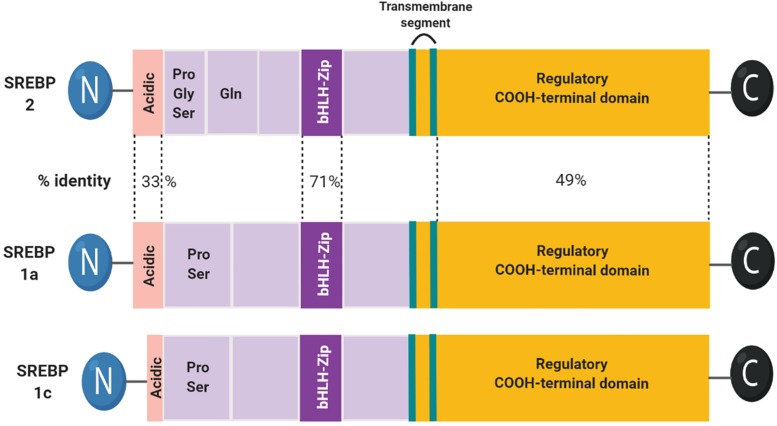
Domain structure of the SREBP family. The structure of SREBP1c is highly similar to SREBP1a. SREBP1c has a shorter transactivation domain in the N-terminus.

SREBPs are bHLH-LZ (basic-helix-loop-helix-leucine zipper) transcription factors, synthesized as inactive precursors bound to ER membranes. Each precursor is organized into three domains: (1) an NH2-terminal domain containing the transactivation domain, a region rich in serine and proline, and the bHLH-LZ region for DNA binding and dimerization; (2) two hydrophobic transmembrane segments projected into the ER lumen; and (3) a COOH-terminal domain (Goldstein et al., 2006; Figure 1).

### Proteolytic Activation of SREBPs

**FIGURE 2:**
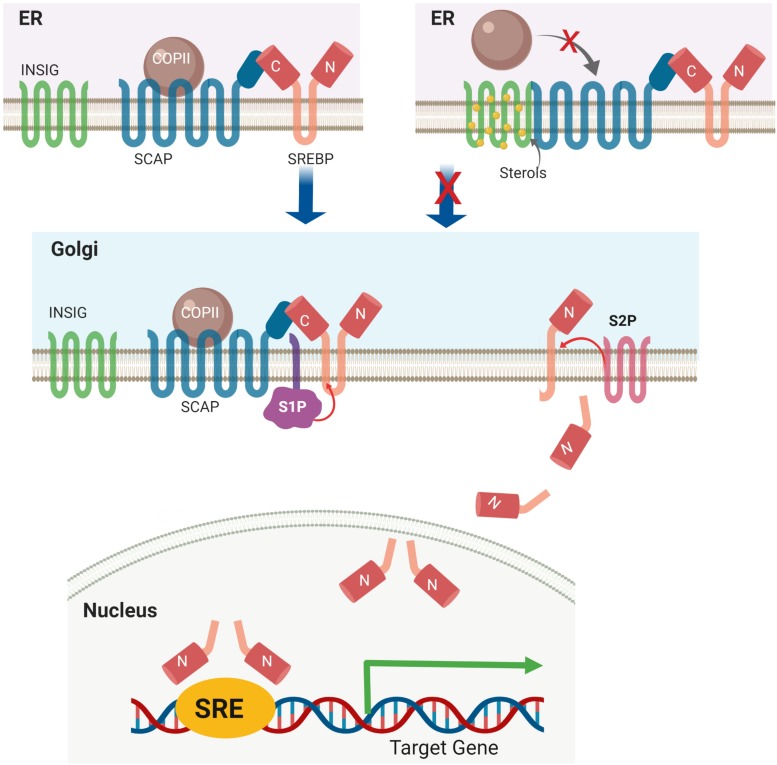
Sterol-mediated proteolytic activation of SREBP. Under basal conditions, the SREBP-SCAP-INSIG complex is retained in the ER membrane. High levels of cellular sterol induce INSIG degradation, followed by translocation of the SREBP-SCAP complex from the ER to the Golgi apparatus. In the Golgi, SREBP is activated via a two-step proteolytic mechanism with S1P and S2P.

Under basal conditions, the C-terminal domain of SREBPs binds to SREBP-cleavage activating protein (SCAP) in the ER membrane. This SREBP-SCAP complex interacts with insulin-induced gene 1 protein (INSIG1) and INSIG2 (Yabe et al., 2002; Yang et al., 2002). In the presence of high cellular cholesterol and oxycholesterol levels, INSIGs become stable and bind to SREBP-SCAP creating a complex retained in the ER membrane. When the level of sterols is low, INSIGs are ubiquitylated by the associated E3 ligases and rapidly degraded (Gong et al., 2006). Meanwhile, the proteolytic activation of SREBP1 is induced by insulin and high glucose and inhibited by polyunsaturated fatty acids (PUFAs) (Nakakuki et al., 2014; Cheng et al., 2015). Insulin-induced Akt activation is shown to decrease INSIG2a protein pools, resulting in increased ER-to-Golgi transport of the SCAP-SREBP1c complex (Yellaturu et al., 2009). SCAP escorts SREBP insertion on ER transport vesicles containing COP II vesicle coat protein. The SREBP-SCAP complex is then transported to the Golgi apparatus (Sun et al., 2005). In the Golgi, the ER luminal loop of SREBP is initially cleaved by site-1 protease (S1P), a membrane-bound serine protease. Subsequent cleavage by site-2 protease (S2P), a Zn^2+^ metalloprotease, generates transcriptionally active N-terminal domains (Ye et al., 2000), which are translocated to the nucleus mediated via importin β (Lee et al., 2003) (Figure 2).

Several kinases have also been reported to be involved in the regulation of proteolytic activation. PAS kinase (PASK), a serine/threonine kinase, is required for the proteolytic maturation of SREBP1c in cultured cells and mice and rat liver. However, the detailed signaling mechanism and its effect on the stability or transcriptional activity of nuclear SREBP1c have not been fully investigated (Wu et al., 2014), whereas AMP-activated protein kinase (AMPK) has been recently identified as an upstream regulator of INSIG1. AMPK phosphorylates INSIG1 (Thr222), abrogates its interaction with E3 ubiquitin ligase gp78, and represses its ubiquitination and degradation. Increased INSIG1 stabilization eventually inhibits the cleavage and processing of SREBP1 (Han et al., 2019).

### Regulation of SREBPs Synthesis and Nuclear Activity

**FIGURE 3:**
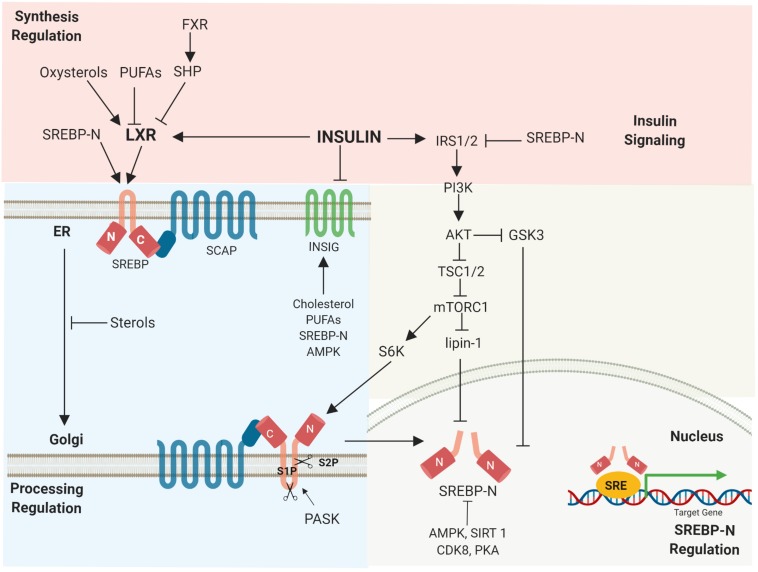
Regulation of SREBPs occurs at various stages. Multiple signals regulate SREBP synthesis, proteolytic activation, transcriptional activity, as well as degradation. Activated, nuclear SREBP is tightly controlled by post-translational modifications.

SREBP1c gene transcription is activated by the liver X receptor (LXR), which is modulated by insulin, PUFAs, and oxysterols. The LXR-retinoid X receptor heterodimer interacts with the LXR-responsive elements located in the SREBP1c promoter, initiating SREBP1c gene transcription (Yoshikawa et al., 2001). Activation of the farnesoid X receptor (FXR) induces small heterodimer partner (SHP) expression, leading to inhibition of LXR and reduced SREBP1c expression (Watanabe et al., 2004; Figure 3).

The transcription of SREBP target genes is tightly regulated by nuclear SREBP stability (Hirano et al., 2001). Mammalian target of rapamycin complex 1 (mTORC1), a major downstream element of insulin-induced phosphatidylinositol 3-kinase (PI3K)/Akt, activates SREBP1 depending on its S6-kinase 1 activity (Düvel et al., 2010). mTORC1 also promotes SREBP via the phosphorylation of lipin1, a phosphatidic acid phosphatase. Dephosphorylated lipin-1 triggers lipin-1 nuclear localization, reduces nuclear SREBP, and alters the localization of SREBP to the nuclear periphery (Peterson et al., 2011). In addition, nuclear SREBPs undergo post-translational modifications. Glycogen synthase kinase 3 (GSK3) phosphorylates nuclear SREBP1a (Ser-434), which serves as a recognition motif for Fbw7 ubiquitin ligases, leading to SREBP1a degradation (Bengoechea-Alonso and Ericsson, 2009). AMPK directly phosphorylates both precursor and nuclear SREBP1c (Ser372), decreasing SREBP1c cleavage and nuclear translocation (Li Y. et al., 2011). NAD^+^-dependent deacetylase Sirtuin 1 (SIRT1) deacetylates and inhibits SREBP1c transactivation by enhancing ubiquitination (Bengoechea-Alonso and Ericsson, 2009; Figure 3).

Furthermore, cyclin-dependent kinase 8 (CDK8) and its regulatory partner cyclin C (CycC) have been identified to cause the phosphorylation of nuclear SREBP1c at a conserved threonine residue (T402), leading to increased nuclear SREBP1c ubiquitination and degradation. Interestingly, CDK8 and CycC are negatively regulated by feeding and insulin, the most widely studied stimuli that promote SREBP1 activation (Zhao et al., 2012). Protein kinase A (PKA) also regulates nuclear SREBP1c stability. Under fasting conditions, glucagon-induced PKA activation stimulates the phosphorylation of SREBP1c (Ser308) (Lee et al., 2014) and SREBP1a (Ser331/332) (Dong et al., 2014). This promotes the sumoylation of SREBP1c at Lys98 by the mammalian protein inhibitor of activated STAT (PIAS)y, a SUMO E3 ligase. Sumoylated SREBP1c is readily degraded by ubiquitination, which leads to decreased hepatic lipid metabolism (Lee et al., 2014).

### Classical DNA Binding Sites of SREBPs

**FIGURE 4:**
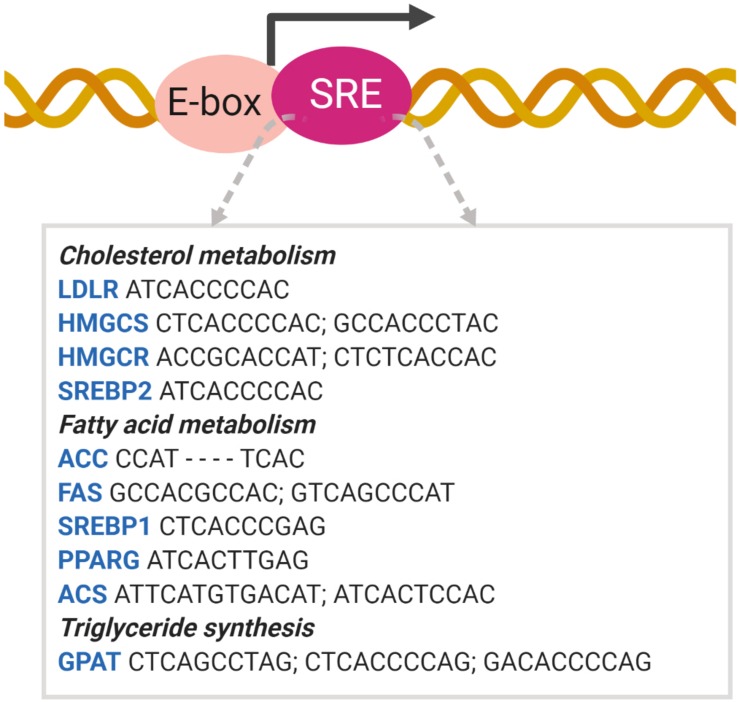
SREBP binds to the SRE promoter. SREBP binding to the SRE promoter activates the transcription of target genes involved in lipid metabolism (Shimano, 2001). ACC acetyl-CoA carboxylase; ACS acetyl-CoA synthetase; GPAT glycerol-3-phosphate acyltransferase; HMGCS HMG-CoA synthase; PPAR peroxisome proliferator-activated receptor.

Intra-nuclear SREBPs were initially found to bind to HMG CoA reductase (HMGCR) and LDL receptor (LDLR) gene promoters, known as sterol response elements (SREs). The original SRE sequence is 5′-ATCACCCCAC-3′ (Hua et al., 1993). In addition to non-palindromic SREs, the SREBPs bind to and activate classic palindromic E-boxes (CAXXTG sequence) containing promoter (Kim et al., 1995). This dual binding specificity allows SREBPs to bind with various cholesterolgenic and lipogenic genes, whose SREs are relatively similar to the original SRE sequence found in the LDLR gene. The sequences of the SREBP binding and activation sites vary considerably and are designated SRE-like sequences (Shimano, 2001; Figure 4).

SREBP1 activates lipogenic genes, whereas SREBP2 is more specific for cholesterolgenic genes. SREBP1a and -2 are equally active for SRE, whereas SREBP1c is inactive. However, only SREBP1a and -1c are active for E-box (Amemiya-Kudo et al., 2000). Due to the high similarity between the N-terminal domains of the three isoforms, all SREBP isoforms can activate each of their target genes but with different efficacies. As SREBP1c contains a shorter transactivation domain, it is weaker than SREBP1a and SREBP2. In contrast, the C-terminal domains of SREBP1 and SREP-2 are relatively less conserved compared to other domains, which may lead to minor differences in the sterol regulation mechanism (Shimano et al., 1997; Pai et al., 1998).

### SREBPs Mediate Renal Lipotoxicity

Altered lipid metabolism has been linked to the progression of both acute and CKDs. Microarray analysis of human kidney biopsies in the Nephroseq database^1^ revealed a higher expression of SREBF1 and SREBF2 in patients with CKD or diabetic kidney disease (DKD) than in normal patients according to the results reported in *Ju CKD Glom* (Ju et al., 2013), *Nakagawa CKD* (Nakagawa et al., 2015), and *Woroniecka Diabetes Glom* (Woroniecka et al., 2011; Na et al., 2015). Furthermore, nutrient and genetic manipulations in experimental animal studies have demonstrated increased SREBP expression, which was associated with renal lipid accumulation, as well as progressive kidney injuries (Table 1).

**TABLE 1:** SREBPs and their target gene expressions mediating renal lipid accumulation and disease progression.

Experimental condition	SREBP in kidney	Expression of target genes	Lipid content in kidney	Renal pathology	Metabolic changes	References
**TYPE 1 DIABETES MELLITUS**						
STZ^∗^-induced SD^∗^ rats	P: ↑1	P: ↑ FAS	TG ↑2-fold	↑ Proteinuria P: ↑ COL4A1, FN1^∗^		Sun et al., 2002
Ins2 Akita mice	P: ↑1, 2		TC^∗^ ↑1.6-fold, TG ↑1.4-fold	↑ Urinary albumin/creatinine P: ↑ COL4A1, FN1^∗^ R: ↑ *Tgfb, Pai1, Vegf*^∗^, *Tnfa* R: ↓ *Synpo*^∗^, *Nphs2*^∗^		Proctor et al., 2006
OVE26 mice	R: ↑1c, 2	R: ↑*Acc, Fas, Hmgcr* R: ↑ *Pparg*		R: ↑ *Fn1*^∗^, *Col4a1, Il6, Tnfa* R: ↓ *Synpo*^∗^, *Nphs2*^∗^		Proctor et al., 2006
**TYPE 2 DIABETES MELLITUS**						
FVB*^*db/db*^*mice	P: ↑ n 1,2	R: ↑ *Fas, Acc, Hmgcr, Ldlr*	TC^∗^ ↑2.2-fold TG ↑2.4-fold	↑ Urinary albumin/creatinine ↑ GBM^∗^ thickness, podocyte foot process length R: ↑ *Tgfb, Pai1, Vegf*^∗^	BW^∗^ TC^∗^ ↑2.1-fold TG ↑1.6-fold	Wang et al., 2005
C57BL/6J mice with HFD^∗^	P: ↑n1,2 R: ↑1a, 1c, 2	R: ↑ *Fas, Acc, Hmgcr*	TC^∗^ ↑1.2-fold TG ↑3-fold	↑ Urinary albumin/creatinine P: ↑ COL4A1, FN1^∗^, PAI1, VEGF^∗^	BW^∗^ TC^∗^ ↑1.4-fold TG ↑1.2-fold Insulin ↑13.4-fold	Jiang et al., 2005c
STZ^∗^-induced SD^∗^ rats fed with HFD^∗^& sucrose diet	P: ↑2, n2 R: ↑2	P: ↑ HMGCR, LDLR		↑ Serum Creatinine, BUN^∗^, Albuminuria, Urinary NGAL^∗^	TC^∗^ ↑6.8-fold TG ↑5-fold LDL^∗^ ↑6.2-fold	Sun et al., 2013
**HYPERTENSION**						
AngII infused SD^∗^ rats	P: ↑1		TC^∗^ ↑1.5-fold TG ↑1.5-fold	P: ↑ TGFB	BP^∗^ TC^∗^ ↑1.3-fold TG ↑2-fold	Saito et al., 2005
**AGING**						
C57BL/6 mice, 23 months vs. 3 months	P: ↑n1, n2 R: ↑1c	P: ↑ HMGCR R: ↑ *Hmgcr, Ldlr*	TC^∗^ ↑3-fold TG ↑3-fold	↑ Urinary albumin/creatinine ↑ GBM^∗^ thickness, podocyte width and effacement P: ↑ COL4A1, FN1^∗^		Jiang et al., 2005a
F344BN rats, 24 months vs. 4 months	P: ↑n1, n2	P: ↑ HMGCR	TC^∗^ ↑1.5-fold TG ↑2.6-fold	↑ Urinary albumin/creatinine P: ↑ COL4A1, FN1^∗^ CTGF, PAI1, VEGF^∗^	TC^∗^ ↑1.5-fold TG ↑2-fold Leptin ↑5.6-fold	Jiang et al., 2005b
SD^∗^ rats, 24 months vs. 6 months	P: ↑1		TG ↑2-fold	↑ Serum Urea, Serum KIM1^∗^ P: ↑ COL1A1, COL4A1, FN1^∗^		Chung et al., 2018
**GENETICALLY MODIFIED MICE**						
Transgenic SREBP1a	P: ↑ 1	R: ↑*Fas, Acc*	TC^∗^ ↑1.2-fold TG ↑2.5-fold	↑ Urinary albumin/Creatinine P: ↑ COL4A1, FN1^∗^ TGFB, VEGF^∗^	Liver TG ↑20-fold TC^∗^ ↑3.5-fold	Sun et al., 2002
SREBP1c knockout			TG ↓ 1.7-fold (vs WT^∗^ in HFD^∗^ group)	R: ↓ *Col4a1, Fn1^∗^, Pai1*, Vegf^∗^(vs WT^∗^ in HFD^∗^ group)	TG ↓↑1.7-fold TC^∗^ ↓1.2-fold (vs WT^∗^ in ND^∗^ group)	Jiang et al., 2005c

*^∗^BP, blood pressure; BUN, blood urea nitrogen; BW, body weight; FN1, fibronectin; GBM, glomerular basement membrane; HFD, high-fat diet; KIM1, kidney injury molecule; LDL, low-density lipoprotein; n, nucleus; ND, normal diet; NGAL, neutrophil gelatinase-associated lipocalin; NPHS2, stomatin family member, podocin; P, protein; R, mRNA; SD rats, Sprague Dawley rats; STZ, streptozotocin; SYNPO, synaptopodin; TC, total cholesterol; VEGF, vascular endothelial growth factor; WT, wild-type.*

The accumulation of non-esterified fatty acids and their metabolites in the kidney leads to cellular dysfunction and death through various mechanisms, such as altered mitochondrial energy coupling, excessive reactive oxygen species generation, and stimulation of ER stress (Murea et al., 2010). Defective fatty acid oxidation (FAO) in renal tubular epithelial cells particularly plays a crucial role in the progression of kidney fibrosis. Genome-wide transcriptome studies of fibrotic human kidneys revealed a lower expression of key enzymes and regulators of FAO (Kang et al., 2015). Kidney biopsies of patients with DKD demonstrated abnormal lipid metabolism, which was significantly correlated with a decline in glomerular filtration rate and kidney inflammation. The genes involved in the FAO pathway, TG hydrolysis, and cholesterol efflux were downregulated, whereas the cholesterol uptake receptor-related gene expression was elevated in the kidneys of human DKD patients (Herman-Edelstein et al., 2014).

### SREBP-Induced Lipotoxicity Also Applies to Other Organs

In addition to kidney diseases, which are the focus of our review, the lipid accumulation induced by SREBP has been demonstrated to aggravate disease progression in other organs, such as the liver and lungs. Increased SREBP activity in the liver causes hepatic steatosis (Shimomura et al., 1999a), that can eventually progress to liver fibrosis and liver failure (Cohen et al., 2009). Specifically in the liver, the synthesis and proteolytic processing of SREBP1c is mainly triggered by insulin (Shimomura et al., 1999b; Brown and Goldstein, 2008). When insulin resistance is evident in peripheral tissues, insulin continues to activate SREBP1c transcription and proteolytic cleavage. Thus, the upregulated nuclear SREBP1c enhances lipogenic gene expression, fatty acid synthesis, and TG accumulation (Shimomura et al., 1999a, 2000). Meanwhile, lipid homeostasis in the lung is precisely regulated to maintain proper lung function, partially regulated by SREBP1c expression. The deletion of SCAP was shown to inhibit SREBP activity in alveolar type 2 cells and enhance neutral lipid accumulation in the lung fibroblasts of fetal and postnatal mice (Besnard et al., 2009). Double Insig1 and Insig2 gene deletions in alveolar type 2 cells activated SREBP1, leading to cholesterol esters and TG accumulation in type 2 epithelial cells and alveolar macrophages. Enhancing lipogenesis in respiratory epithelial cells resulted in lipotoxicity-related lung inflammation, airspace abnormalities, and tissue remodeling (Plantier et al., 2012). Aside from this lipid-dependent pathway, to our knowledge, there has been no study in the liver and lungs elucidating the mechanism of SREBP in mediating fibrotic signaling through a lipid-independent pathway.

## Srebp as a Pro-Fibrotic Mediator in the Kidney

Although most of the early works have already shown the role of SREBP in lipotoxicity-induced progressive tissue injuries and fibrosis development, a plausible role for SREBP in directly mediating the activation of fibrotic signaling has been underscored. SREBP was initially reported to induce extracellular matrix (ECM) gene transcription in fibroblasts. The promoter of collagen (col) VI (α1) contains a growth arrest-responsive region (GARR), comprised of repeat GA-box motifs (GGGGAGGG). In NIH3T3 fibroblasts cultured in serum-free medium, SREBP bound to GARR and induced Col VI (α1) transcription (Ferrari et al., 2004). Plasminogen activator inhibitor 1 (PAI1), a serine protease with pro-fibrotic properties, has been also identified as a transcriptional target of SREBP1c in 3T3-L1 adipocytes (Lay et al., 2002). Following these precedent reports, a series of studies in the kidney were performed to further demonstrate a transcriptional effect of SREBP on the fibrotic signaling pathway.

### Non-sterol-mediated SREBP Activation

As described in the previous section, the activation of SREBP is classically regulated by cellular sterol levels. Other tissue-injury stimuli, such as mechanical cues (Liu et al., 2002; Lin et al., 2003) and high glucose (HG) (Hasty et al., 2000; Guillet-Deniau et al., 2004; Uttarwar et al., 2012), have been reported to induce SREBP activation, as well. However, the signaling mechanism of non-sterol-mediated SREBP activation appears to depend on the type of stimulus. SREBP1 activation in endothelial cells exposed to shear stress requires signaling via β1 integrin, focal adhesion kinase, and c-Src (Liu et al., 2002). The shear stress activates SREBP2 in vascular endothelial cells via RhoA/Rho-kinase signaling. The Rho-ROCK-LIMK-cofilin pathway enhances the actin assembly needed for SREBP transport from the ER to Golgi (Lin et al., 2003). In recent work, ECM stiffening and geranylgeranylated RhoA-dependent acto-myosin contraction were also shown to activate AMPK, resulting in inhibition of SREBP1 activation (Bertolio et al., 2019).

Glucose has been shown to activate SREBP1 in renal tubular epithelial cell lines (Jun et al., 2009). Several studies have suggested that epidermal growth factor receptor (EGFR)/PI3K/Akt (Wu et al., 2007, 2009) and RhoA/Rho-kinase (Peng et al., 2008) signaling mediate HG-induced TGFβ upregulation and ECM accumulation in the kidney. A mechanistic study with primary rat mesangial cells (MCs) confirmed the role of these signaling in mediating HG-induced SREBP1 activation, which leads to TGFβ activation. S1P and SCAP were required for SREBP1 activation, as it was blocked by chemical inhibitors of S1P and SCAP (Wang T. N. et al., 2015). Despite the technical limitations associated with the differentiation of the two isoforms of SREBP1, these studies suggested that multiple factors activate SREBP1, which is associated with kidney injuries. By utilizing more recent and sensitive techniques, it remains challenging to differentiate the SREBP isoforms activated by specific stimuli and the respective downstream signaling in the kidney.

### SREBP Regulates TGFβ Activity

Particularly in the kidney, SREBP has then been shown to be activated by several non-sterol stimuli and to contribute to the activation of fibrotic signaling, i.e., TGFβ, a multifunctional cytokine that classically plays a major role in progressive kidney fibrosis (Sureshbabu et al., 2016).

#### SREBP Induces TGFβ Transcriptional Activity

HG increased TGFβ activity via SREBP1 activation in primary rat MCs. As early as 30 min and persisting up to 6 h, 30 mM HG activated SREBPs based on the detection of its mature form (nSREBP1), whereas SREBP2 was not activated. HG promoted SREBP1 binding to the TGFβ promoter, which contains a putative SREBP1 binding site, SRE. This binding was halted by fatostatin and dominant-negative SREBP1a Y335A. Connective tissue growth factor (CTGF) was detected as the downstream target of TGFβ activation. Interestingly, TGFβ promoter analysis revealed that a potential SRE site was within the first 100 base pairs of the start codon. The site is located apparently in close proximity to the Sp1 site, a well-known coactivator of SREBP (Uttarwar et al., 2012).

Angiotensin (Ang)-II also stimulated SREBP1-induced TGFβ signaling in primary rat MC cultures. Treatment with 100 nM Ang II induced SREBP1 maturation, activated SREBP1 as detected by responsive SRE, and increased the SREBP downstream target, fatty acid synthase (FAS), leading to lipid accumulation. Then, Ang II activated two parallel signals essential for TGFβ promoter activation, ER stress-induced SREBP1 activation and EGFR-mediated activation of co-transcription factor Sp1. ER stress and SREBP1 activation were detected in the glomeruli of Ang II infused mice. SREBP1 inhibition by fatostatin prevented Ang II-induced TGFβ upregulation and ECM accumulation. Mechanistically, Ang II-induced SREBP1 activation in MC culture required angiotensin (AT)1 receptor/PI3K/Akt signaling (Wang T. N. et al., 2015). PI3K and Akt are the downstream signaling molecules of AT1 receptor via coupling to G-proteins that positively regulate SREBP activation (Yellaturu et al., 2009; Uttarwar et al., 2012). Ang II-induced ER stress, exhibited by increased p-eIF2α and GRP78 expression, also activated SREBP1 in MC culture. Inhibition of ER stress or SREBP1 prevented Ang II-induced SREBP1 binding to the TGFβ promoter (Wang T. N. et al., 2015). Another study showed that ER stress decreased INSIG via eIF2α translational inhibition, resulting in the proteolytic activation of SCAP/SREBP. GRP78 retained SCAP/SREBP1 in the ER via direct interaction, which was disrupted during ER stress (Colgan et al., 2011). However, this Ang II/ER stress/SREBP activation needs to be confirmed in experimental animal models to provide stronger evidence. As Ang II might also induce ER stress in podocytes (Ha et al., 2015) and proximal tubular epithelial cells (Wang J. et al., 2015), other studies with these kidney cell subtypes are needed, as well. EGFR may be an important second signal in TGFβ upregulation via activation of the SREBP1 co-transcription factor Sp1. However, EGFR was not required for either SREBP1 activation or TGFβ promoter binding by SREBP1 (Wang T. N. et al., 2015).

A recent study suggested the role of lysophosphatidic acid (LPA) in SREBP-induced TGFβ activity (Li et al., 2017). LPA is a small, ubiquitous phospholipid that mediates pro-inflammatory and pro-fibrotic signaling in the kidney (Zhang et al., 2017). TGFβ and Smad-2/3 phosphorylation were upregulated in the renal cortex of db/db mice and SV40 MES13 MCs stimulated with LPA. LPA bound to its receptor and activated PI3K/Akt phosphorylation, leading to phosphorylation of GSK3β at Ser9. The inactive, phosphorylated GSK3β decreased SREBP1 degradation, induced its nuclear translocation, and eventually triggered TGFβ transcriptional activity. Inhibition of either LPA receptor or SCAP by fatostatin significantly decreased TGFβ signaling and ECM accumulation in mesangial cells (Li et al., 2017).

One should be aware that the cited evidence linking SREBP and TGFβ activation was derived from different experimental conditions or biological contexts. Those studies utilized EMSA and ChiP to discover the binding site motif, but the methods were limited by the skewed distribution of the genomic sequence and the inability to distinguish direct and indirect binding of the transcription factor (Lambert et al., 2018). A combination of methods with different throughput and information content, e.g., ChiP combined with SELEX, protein binding microarray/PBM, and MITOMI, are ideally necessary to determine and validate the precise genomic binding site of the transcription factor and how the transcription factor binding ultimately relates to the regulation of transcription (Geertz and Maerkl, 2010; Lambert et al., 2018).

#### A Vicious Cycle of SREBP1 and TGFβ Activation

**FIGURE 5:**
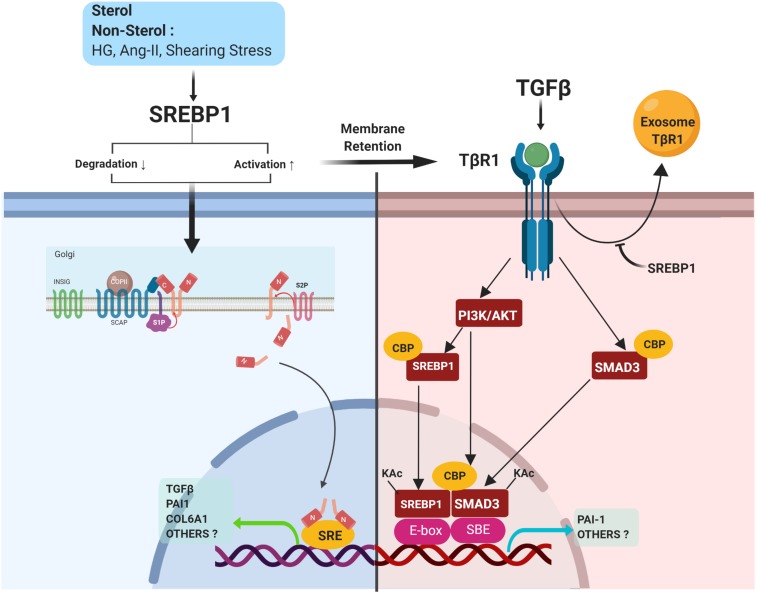
SREBP1 directly activates pro-fibrotic signaling. SREBP activation is induced by either sterol or non-sterol stimuli, resulting in fibrotic signaling. In the kidney, SREBP1 regulates TGFβ activity via 1) induction of TGFβ transcriptional activity, 2) a positive feedback loop with TGFβ/Smad3 signaling, and 3) prevention of exosomal degradation of the TGFβ-receptor.

Active SREBP1a, but not SREBP1c, activates the TGFβ-responsive reporter plasmid p3TP-lux carrying the promoter region of PAI1 (Chen et al., 2014). Due to its additional N-terminal residues, SREBP1a can bind to co-transcriptional factors, such as CREB-binding protein (CBP), making it a more potent transcriptional factor than SREBP1c (Toth et al., 2004). However, TGFβ activates not only its canonical pro-fibrotic downstream target, Smad3, but also non-canonical SREBP1, which requires SCAP, S1P, and P13K/Akt signaling (Figure 5). TGFβ-induced PI3K/Akt signaling acts as a critical regulator of Smad3-CBP interaction and Smad3 acetylation, which results in the upregulation of PAI1 expression (Das et al., 2008). Moreover, TGFβ-induced PI3K/Akt signaling stimulated acetylation of SREBP1a in the lysine residue (K333) by the acetyltransferase CBP, which enhanced the nuclear stability of SREBP1a. The active SREBP1a was further bound to E-box, which was adjacent to the Smad-binding element (SBE). TGFβ, interestingly, induced a direct association between Smad3 and acetylated SREBP1a in this adjacent area and the interaction between these two transcription factors was crucial in regulating the transcriptional activity of TGFβ (Chen et al., 2014). Along with previous observations suggesting that SREBP1 mediates the upregulation of TGFβ transcriptional activity (Uttarwar et al., 2012; Wang T. N. et al., 2015; Li et al., 2017), a positive feedback loop may exist in which SREBP1 exacerbates both TGFβ transcriptional activity and the response, to trigger progressive fibrosis (Figure 5).

#### SREBP Prevents Exosomal Degradation of TGFβ Receptor (TβR)

As discussed above, SREBP1 coordinates TGFβ signaling via its interaction with Smad3 (Chen et al., 2014). The activation of Smad2/3 can be controlled at various levels, for example, via the turnover of TβRI and TβRII (Huang and Chen, 2012). SREBP1 inhibition by either fatostatin or SREBP1 siRNA decreased TβRI expression and halted TGFβ/Smad3 signaling. However, SREBP1 did not regulate TβRI via transcription, proteasomal/lysosomal degradation, or proteolytic cleavage (Van Krieken et al., 2017).

Lipid rafts and caveolar endocytosis have been associated with the downregulation of TβRI (Huang and Chen, 2012; Lan and Chung, 2012). Cyclodextrin-induced lipid raft disruption prevented TβRI decreases, suggesting that SREBP1 regulated the cell surface expression of TβRI in a lipid-raft dependent manner. However, TβRI expression in MCs was found to be independent of caveolae since SREBP1 inhibition still induced TβRI downregulation in caveolin-1 knockout MCs. SREBP1 may act as an important cell surface retention factor for TβRI by preventing its secretion in the exosome (Figure 5). SREBP1 inhibition induced TβRI secretion into exosomes, which has been implicated in intracellular organelle transfer (Van Krieken et al., 2017). However, this study did not exclude the plausible re-fusion of exosomes containing TβRI in the adjacent MCs.

## Potential Targets of SREBPs: Beyond Lipid Metabolism

In a study by Seo et al. (2009), the genome-wide analysis of SREBP1 binding in mouse liver chromatin revealed a preference for proximal binding of the promoter to a new motif (5′-ACTACANNTCCC-3′). Since then, many putative target genes of SREBPs have been shown to act beyond lipid metabolism, such as in autophagy (Seo et al., 2009), ER stress (Sanchez-Alvarez et al., 2014), as well as metabolic circadian rhythm (Gilardi et al., 2014).

### Identification of Non-lipogenic Target Genes of SREBPs

**FIGURE 6:**
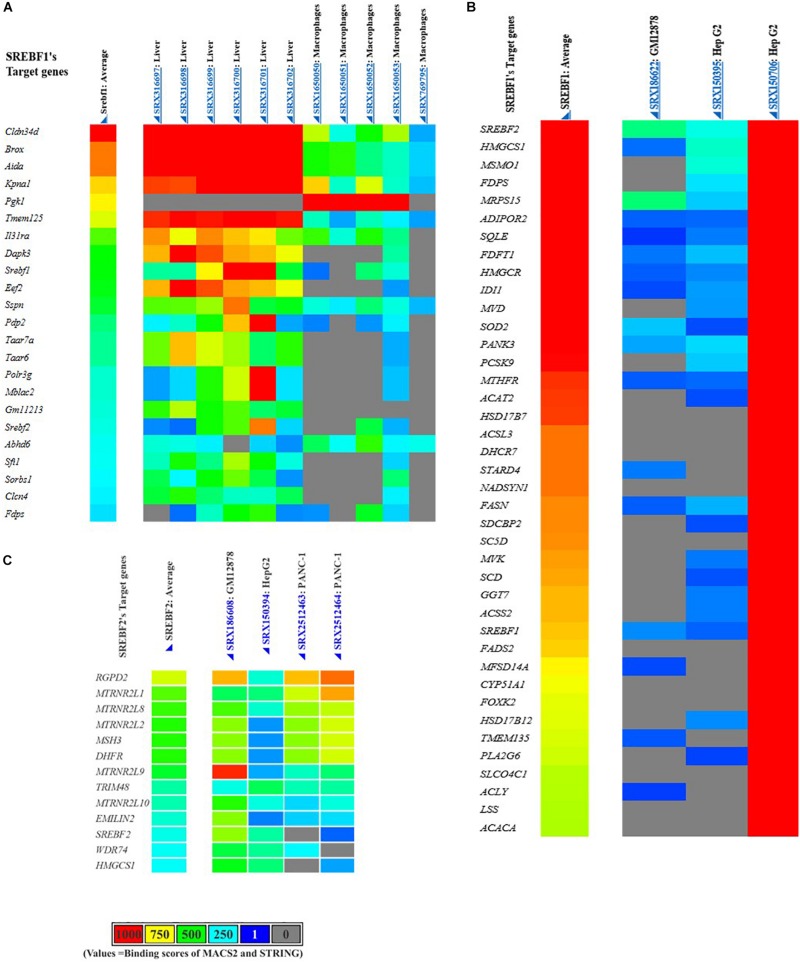
Putative SREBFs target genes generated from the ChIP-Atlas database. *Mus musculus* or mouse SREBF1 target genes **(A)**, *Homo sapiens* or human SREBF1 **(B)**, and SREBF2 target genes **(C)**.

Having been identified to promote the transcriptional activity of TGFβ, we wondered whether other pro-fibrotic mediators could also be targets of SREBP regulation. Here, the potential SREBF target genes were identified using a data-mining suite powered by full integration of public chromatin immunoprecipitation (ChIP)-sequencing data, namely ChIP-Atlas^2^. The transcription regulatory peaks were examined from around ± 5 kb of the transcription start sequence of the coding genes (Figure 6). However, it should be noted that the genes listed as target genes are not necessarily functional targets of a given transcriptional regulator, suggesting that the actual regulation of potential target genes should be experimentally verified (Oki et al., 2018).

### Potential Targets of SREBPs for the Regulation of Fibrosis Development

As summarized in Table 2, SREBFs are predicted to regulate various non-lipogenic genes in diverse tissues and cell lines. Among those genes, we discuss several SREBP target genes that are plausibly involved in the pathogenesis of tissue fibrosis. These target genes would be interesting to be directly investigated in an experimental disease model of either kidney or other organs.

**TABLE 2:** Lipid and non-lipid targets of SREBF genes generated from the Chip-Atlas database (https://chip-atlas.org/).

Biological Function	Target Genes
**SREBF1 MUS MUSCULUS**	
**Lipid biosynthesis**	*Srebf1; Srebf2*
**Non-lipid targets**	
Antiport system	*Clcn4*
Associated protein of endosomal sorting complex	*Brox*
Cytoskeleton and ECM	*Sspn*
RNA polymerase activity	*Polr3g*
Essential factor for protein synthesis	*Eef2*
Cell matrix adhesion	*Sorbs1; Cldn34d*
Glycolytic enzyme	*Pgk1*
Hydrolase activity	*Mblac2*
Induction of apoptosis	*Dapk3*
Interleukin-31 receptor signaling	*Il31ra*
Lipase activity	*Abhd6*
Nuclear protein import	*Kpna1*
Phosphatase activity	*Pdp2*
Structural protein of centrosome	*Sfi1*
Trace-amine receptor	*Taar6; Taar7a*
Transmembrane protein	*Tmem125*
Ventralizing factor in embryogenesis	*Aida*
**SREBF1 HOMO SAPIENS**	
**Lipid biosynthesis**	*ACACA, ACAT2, ACLY, ACSL3, ACSS2, CYP51A1, DHCR7, FADS2, FAS, FDFT1, FDPS, HMGCR, HMGCS1, HSD17B12, HSD17B7, IDI1, LSS, MSMO1, MVD, MVK, SC5D, SCD, SQLE, SREBF1, SREBF2, STARD4*
**Non-lipid targets**	
Adiponectin signaling	*ADIPOR2*
Biosynthesis of coenzyme A	*PANK3*
Cell matrix adhesion	*SDCBP2*
Glutathione metabolism	*SOD2*
Mitochondrial translation	*MRPS15*
Peroxisome organization, mitochondrial fission	*TMEM135*
Phospholipase activity	*PLA2G6*
Transcriptional regulator	*FOXK2*
Transmembrane transporter	*SLCO4C1*
**SREBF2 HOMO SAPIENS**	
**Lipid biosynthesis**	*SREBF2; HMGCS1*
**Non-lipid targets**	
Anti-apoptotic	*MT-RNR2L*
*De novo* synthesis of purines, thymidylic acid	*DHFR*
DNA mismatch repair	*MSH3*
ECM constituent	*EMILIN2*
Ribosome biogenesis	*WDR74*

#### Claudin 34D (*CLDN34D*)

Claudins are a family of tight-junction membrane proteins that have not been clearly elucidated in the kidney. Genetic mutations in claudin-16 and -19 cause familial hypomagnesemia and hypercalciuria with nephrocalcinosis, whereas polymorphisms in claudin-14 are associated with the risk of kidney stones (Li J. et al., 2011; Yu, 2015). In the lung, the disruption of claudin-18 expression stimulates pulmonary fibrosis or chronic obstructive pulmonary disease (Schlingmann et al., 2015). Furthermore, claudin modification has been established in several cancers, including gastric cancer, via the activation of epithelial-to-mesenchymal transition (EMT), a mechanism that is also involved in the progression of fibrosis (Rendon-Huerta et al., 2013).

#### Syndecan Binding Protein (Syntenin) 2 (*SDCBP2*)

Syntenin was initially identified as a protein linking syndecan-mediated signaling to the cytoskeleton (Grootjans et al., 1997). Syntenin positively regulates TGFβ-mediated Smad activation and EMT by preventing the caveolin-1-mediated internalization of TβRI (Hwangbo et al., 2016). Thus, this adapter protein might contribute to tissue fibrosis.

#### Elastin Microfibril Interfacer 2 (*EMILIN2*)

EMILIN is an elastic fiber-associated glycoprotein (Doliana et al., 2001). In the kidney, it is localized to the glomeruli and occurs predominantly in mesangial cells (Sterzel et al., 2000). EMILIN2 binds to the TNF-related apoptosis-inducing ligand (TRAIL) death receptor (DR) 4 and partially with DR5 to activate the extrinsic apoptotic pathway. EMILIN2 knockdown results in enhanced cell survival and its overexpression triggers massive apoptosis (Mongiat et al., 2007). These additional mechanisms of ECM in modulating cell survival require further exploration in kidney diseases.

#### Death Associated Protein Kinase 3 (*DAPK3*)

DAPK is a calcium/calmodulin-regulated serine/threonine kinase that mediates cell death. Deletion of the kinase domain in DAPK attenuates tubular cell apoptosis in renal ischemia-reperfusion injury (Kishino et al., 2004). DAPK3 induces apoptosis or autophagy with or without caspase proteins. It also mediates inflammatory signals L13a (ribosome protein), ERK, and interferon-γ-activated inhibition of translation (Elbadawy et al., 2018). However, it is unclear whether DAPK3 is a potential target for kidney protection against cell death.

#### Transmembrane Protein 135 (*TMEM135*)

TMEM135 is an LXR-inducible regulator of peroxisome catabolic and anabolic processes mediated via the auxiliary matrix protein import pathway. It also regulates mitochondrial dynamics to protect the retina against oxidative stress and progressive retinal aging (Lee et al., 2016). Overexpression of TMEM135 increases mitochondrial fragmentation, as well as collagen accumulation and hypertrophy in the heart (Lewis et al., 2018). However, the role of TMEM135 in modulating mitochondria or peroxisomal function in kidney tissue remains elusive.

## Future Directions and Conclusion

Given the importance of SREBP in mediating lipid biosynthesis, which converges with various pathological signaling mechanisms, targeting SREBP is an important pharmacological strategy to attenuate the progression of kidney diseases. The available small-molecule inhibitors of SREBP activation have been comprehensively reviewed elsewhere (Watanabe and Uesugi, 2013). However, fatostatin is the only SREBP inhibitor extensively studied in kidney diseases. It inhibits the ER-Golgi translocation of SREBPs by binding to SCAP at a site distinct from the sterol-binding domain. Decreased SREBP maturation attenuates the progression of tubulointerstitial fibrosis induced by unilateral obstructive injury (Mustafa et al., 2016) and kidney injury in hypertensive mice (Wang T. N. et al., 2015). In type 1 diabetic mice, 12-week fatostatin treatment blocked renal SREBP1 and SREBP2 expression. However, hyperfiltration, albuminuria, and kidney fibrosis were not attenuated in the diabetic mice. Non-diabetic mice treated with fatostatin exhibited hyperfiltration and increases in glomerular volume to levels seen in diabetic mice, which were associated with increased kidney inflammation and a trend toward fibrosis (Van Krieken et al., 2018). Thus, the efficacy of other SREBP inhibitors and the specific roles of SREBP in the pathogenesis of DKD and CKD remain to be investigated.

**FIGURE 7:**
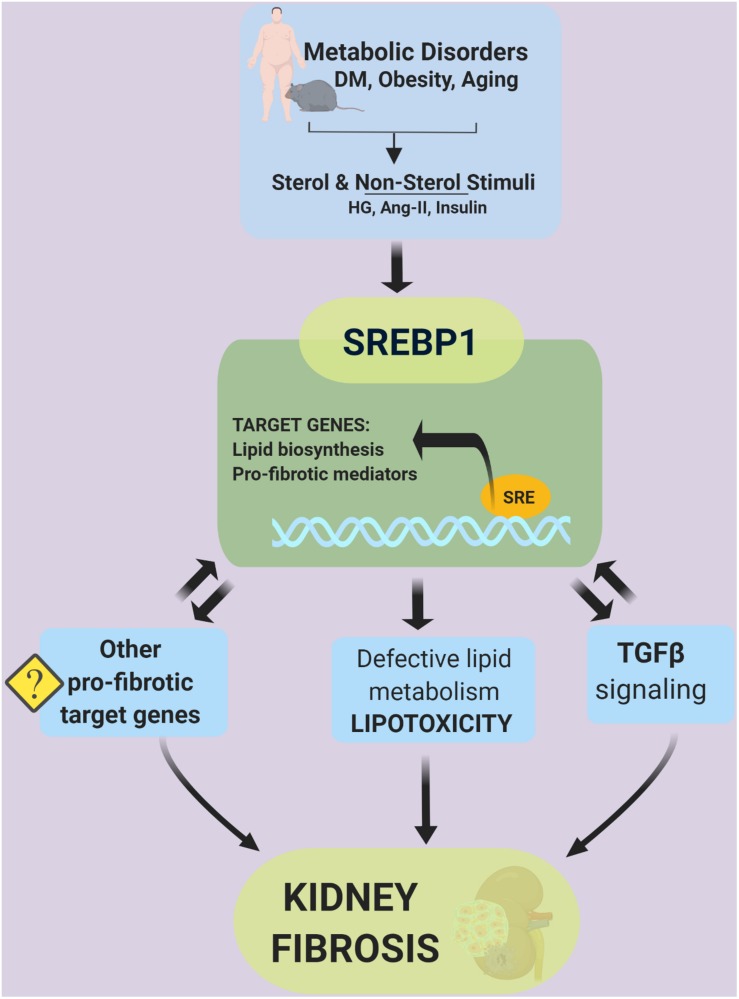
SREBPs mediate kidney fibrosis via lipid-dependent and -independent pathways.

Genome-wide analysis, *in vitro*, and *in vivo* studies have demonstrated the versatility of SREBPs in mediating diverse biological processes. Particularly in the kidney, SREBP1 acts as an activator of pro-fibrotic signaling by binding to the promoter area of fibrosis-related genes, i.e., TGFβ. The precise elucidation of non-lipid and direct or indirect targets of SREBPs that mediate the development of fibrosis remains a challenge. Emerging data suggest that continued investigation of the SREBP pathway and the discovery of its small molecule inhibitors will facilitate the amelioration of kidney disease via lipid-dependent and -independent pathways (Figure 7).

## Author Contributions

DD conceived and wrote the manuscript, and designed the figures. DK and HH provided critical revisions of the manuscript. HH made the final approval of the version to be published.

## Conflict of Interest

The authors declare that the research was conducted in the absence of any commercial or financial relationships that could be construed as a potential conflict of interest.

## Footnotes

1 http://www.nephroseq.org/

2 https://chip-atlas.org/

## APPENDIX

**Abbreviations:** ABHD6, abhydrolase domain containing 6; ACACA, acetyl-Coa carboxylase alpha; ACAT, acetyl-Coa acetyltransferase; ACC, acetyl-CoA carboxylase; ACLY, ATP citrate lyase; ACOX, acyl-CoA oxidase; ACS, acetyl-CoA synthetase; ACSL, acyl-CoA synthetase long-chain family member; ACSS, acyl-CoA synthetase short-chain family member; ADIPOR2, adiponectin receptor; AIDA, axin interactor, dorsalization associated; AMPK, AMP-activated protein kinase; Ang II, angiotensin II; AT1, angiotensin II type 1; BROX BRO1, domain and CAAX motif containing; CBP, CREB-binding protein; CDK8, cyclin-dependent kinase 8; ChIP, chromatin immunoprecipitation; CKD, chronic kidney disease; CLCN4, chloride voltage-gated channel 4; CLDN34D, claudin 34D; COL, collagen; CPT, carnitine palmitoyltransferase; CTGF, connective tissue growth factor; CYP51A1, cytochrome P450 family 51 subfamily A member 1; DAPK3, death-associated protein kinase 3; DHCR7, 7-dehydrocholesterol reductase; DHFR, dihydrofolate reductase; DKD, diabetic kidney disease; ECM, extracellular matrix; EEF2, eukaryotic translation elongation factor 2; EMILIN2, elastin microfibril interfacer 2; EMT, epithelial-to-mesenchymal transition; ER, endoplasmic reticulum; FADS2, fatty acid desaturase; FAO, fatty acid oxidation; FAS, fatty acid synthase; FDFT1, farnesyl diphosphate farnesyl transferase; FDPS, farnesyl diphosphate synthase; FOXK2, forkhead box K2; FXR, farnesoid x receptor; GARR, growth arrest-responsive region; GM11213, predicted gene 11213; GPAT, glycerol-3-phosphate acyltransferase; GSK, glycogen synthase kinase; HG, high glucose; HMGCR, 3-hydroxy-3-methylglutaryl-Coa reductase; HMGCS, 3-hydroxy-3-methylglutaryl-CoA synthase; HNF4 α, hepatocyte nuclear factor-4 α; HSD17B, hydroxysteroid 17-beta dehydrogenase; IDI1, isopentenyl-diphosphate delta isomerase; IL31RA, interleukin 31 receptor A; INSIG, insulin-induced gene; KPNA1, karyopherin subunit alpha 1; LDLR, LDL receptor; LPA, lysophosphatidic acid; LSS, lanosterol synthase; LXR, liver X receptor; LXRE, LXR-responsive elements; MBLAC2, metallo-beta-lactamase domain containing 2; MC, mesangial cell; MEF, mouse embryonic fibroblast; MRPS15, mitochondrial ribosomal protein S1; MSH3, MutS homolog; MSMO1, methylsterol monooxygenase; mTORC1, mammalian target of rapamycin complex 1; MT-RNR2L, MT-RNR2 like; MVD, mevalonate diphosphate decarboxylase; MVK, mevalonate kinase; PAI1, plasminogen activator inhibitor 1; PANK3, pantothenate kinase; PCSK9, proprotein convertase subtilisin/kexin type 9; PDP2, pyruvate dehydrogenase phosphatase catalytic subunit 2; PGC1 α, proliferator-activated receptor-gamma coactivator 1; PGK1, phosphoglycerate kinase 1; PI3K, phosphatidylinositol 3-kinase; PKA, protein kinase A; PLA2G6, phospholipase A2 group 6; POLR3G, RNA polymerase 3 subunit G; PPAR, peroxisome proliferator-activated receptor; PUFAs, polyunsaturated fatty acids; S1P, site-1 protease; S2P, site-2 protease; SCAP, SREBP cleavage-activating protein; SCD, stearoyl-CoA desaturase; SDCBP2, syndecan binding protein; SFI1, SFI1 centrin binding protein; SIRT1, sirtuin 1; SLCO4C1, solute carrier organic anion transporter family member 4C1; SOD2, superoxide dismutase; SORBS1, sorbin and SH3 domain containing 1; SQLE, squalene epoxidase; SRE, sterol response element; SREBF, sterol regulatory element-binding transcription factor; SREBP, sterol regulatory element-binding protein; SSPN, sarcospan; STARD4, StAR-related lipid transfer domain containing; TAAR, trace amine-associated receptor; TC, total cholesterol; TG, triglyceride; TGF β, transforming growth factor β; TMEM, transmembrane protein; TSC1/2, tuberous sclerosis complex ½; T β RI, TGF β receptor I; WDR74, WD repeat domain 74.
